# Transplacental transmission of tick-borne *Babesia microti* in its natural host *Peromyscus leucopus*

**DOI:** 10.1186/s13071-018-2875-8

**Published:** 2018-05-04

**Authors:** Danielle M. Tufts, Maria A. Diuk-Wasser

**Affiliations:** Ecology, Evolution, and Environmental Biology Department, Columbia University, New York, NY 10027 USA

**Keywords:** *Ixodes scapularis*, Rodent, Babesiosis, Emerging disease, Congenital transmission

## Abstract

**Background:**

*Babesia microti* is an emerging tick-borne pathogen and the causative agent of human babesiosis. Mathematical modeling of the reproductive rate of *B. microti* indicates that it cannot persist in nature by horizontal tick-host transmission alone. We hypothesized that transplacental transmission in the reservoir population contributes to *B. microti* persistence and emergence in North American rodent populations.

**Methods:**

*Peromyscus leucopus* were collected from Connecticut and Block Island, Rhode Island and analyzed using a highly specific quantitative PCR (qPCR) assay for infection with *B. microti*.

**Results:**

In April, 100% (*n* = 103) of mice were infected with *B. microti*. Females exhibited significantly higher parasitemia than their offspring (*P* < 0.0001) and transplacental transmission was observed in 74.2% of embryos (*n* = 89). Transplacental transmission of *B. microti* is thus a viable and potentially important infectious pathway in naturally infected rodent species and should be considered in future theoretical and empirical studies.

**Conclusions:**

To our knowledge, this study is the first to report transplacental transmission of *B. microti* occurring in its natural reservoir host, *P. leucopus,* in the United States and the only study that provides a quantitative estimate of parasitemia. This vector-independent pathway could contribute to the increased geographic range of *B. microti* or increase its abundance in endemic areas.

**Electronic supplementary material:**

The online version of this article (10.1186/s13071-018-2875-8) contains supplementary material, which is available to authorized users.

## Background

*Babesia microti* (Apicoplexa: Sporozoea) is a zoonotic intraerythrocytic apicomplexan parasite and is responsible for almost all cases of human babesiosis in the United States [1]. Human babesiosis is an emerging tick-borne disease that shares the same vector, the blacklegged tick (*Ixodes scapularis*), and dominant reservoir host, the white-footed mouse (*Peromyscus leucopus*), as the causative agent of Lyme disease, the spirochete *Borrelia burgdorferi* [2]. During the last 20 years, human babesiosis has spread in the United States [3, 4] following a similar trajectory to that of Lyme disease, although with a temporal lag [5–7]. Factors accounting for the delayed spread of babesiosis compared to Lyme disease include lower fitness in the enzootic cycle because of lower transmission from infected host to tick and lower trans-stadial transmission, greater asymptomatic infection in humans, insufficient physician awareness, and under-diagnosis in non- or newly-endemic areas [1, 5, 6].

An integrated measure of *B. microti* fitness (the basic reproductive number, *R*_0_) was estimated to be lower than the threshold for pathogen persistence (*R*_0_ < 1) under ecological conditions identified in long-term field studies [6, 8] implying that emergence of this pathogen should be unlikely in nature. However, despite the low predicted *R*_0_, *B. microti* has not only persisted in multiple locations with high infection prevalence in ticks regionally [5, 9–11], but it is also geographically expanding in the Northeast and upper Midwest regions of the USA [6]. This paradoxical finding suggests additional mechanism(s) enhancing *B. microti* transmission and persistence in the enzootic cycle may be occurring. Previous field studies reported higher average infection prevalences of *B. burgdorferi* (22.37%) compared to *B. microti* (9.53%) in nymphal *I. scapularis* ticks throughout the New England area [6]. However, very little is known about the *B. microti* infection status of *P. leucopus* in nature.

The aims of this research were to (i) determine if transplacental transmission of *B. microti* occurs in naturally infected, wild reservoir *P. leucopus* mice and (ii) quantify the level of transplacental transmission of host populations in *B. microti*-endemic coastal New England. As a comparison for early season infection prevalence we also screened for the presence of *B. burgdorferi* in the same host population; *B. burgdorferi* is not known to be transmitted transplacentally [12, 13].

## Methods

### Study site and animals

Adult *Peromyscus leucopus* were collected from two locations in Connecticut [Lake Gaillard (LG) 41°22'25.3"N, 72°46'43"W and Old Lyme (OL) 41°22'21.5"N, 72°20'37.6"W] and two locations on Block Island, Rhode Island [North Island (NI) 41°12'36.4"N, 71°34'18.8"W and Rodman’s Hollow (RH) 41°09'25.2"N, 71°35'22.9"W] for two trapping sessions from April 26 - May 2 (hereafter referred to as April) and July 23–31, 2016. In each trapping session, animals were trapped for two consecutive nights using Sherman live traps (7.62 × 8.89 × 22.86 cm; H.B. Sherman Traps, Inc. Tallahassee, FL) baited with peanut butter, oats, and sunflower seeds. Traps were arranged in nine 200 m transects with one trap placed every 10 m for a total of 180 traps at each location, except for NI where 100 m transects were used for a total of 90 traps.

Animals were removed from traps, morphological characteristics (age, sex, weight, body measurements, etc.) were collected, and attached larval and nymphal ticks were counted and removed from the ears and body. All animals were euthanized with an overdose of isoflurane and necropsied immediately in the field. All organs and tissues were preserved in liquid nitrogen, blood samples were dried on Whatman FTA cards (Fisher Scientific, Pittsburg, PA, USA), and the carcasses were wrapped in Whirl-Pak bags (Whirl-Pak®, Nasco, Fort Atkinson, WI, USA) and frozen in liquid nitrogen; embryos of pregnant females remained within the carcass. All samples were transferred to -80 °C for long term storage upon return to the laboratory. All animal procedures were in accordance with guidelines approved by the Columbia University Institutional Animal Care and Use Committee (IACUC no. AC-AAAL3656).

### Embryo necropsy

**Fig. 1:**
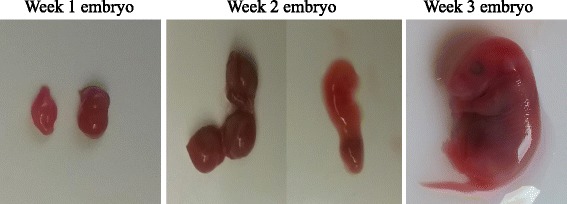
Pictures of embryos in the three stages of development, categorization was based on size and developmental characteristics of each embryo. Week 1 embryos were very small with little to no distinguishing characteristics. Week 2 embryos were larger and an embryo could be removed from its location in the uterus, a distinctive eye-spot was also observed. Week 3 embryos were large with almost fully developed characteristics

To ensure no cross contamination of maternal blood occurred during the necropsy of embryos from the female’s body cavity, a new pair of autoclaved dissecting utensils were used for each adult female. Utensils were soaked in a 10% bleach solution and then in 70% ethanol for at least 2 min between sampling embryos from the same adult female. All embryos were removed from the body cavity of the female, washed with a saline solution, photographed, immediately cut into sections around each embryo, and then transferred to a separate sterile Petri dish. Embryos were found in various stages of development. Because the gestation time of *P. leucopus* is 22–28 days, embryos were separated into three developmental age categories (Week 1, Week 2, Week 3; Fig. 1) based on the size and the presence or absence of various developmental characteristics for each embryo (i.e. eye spot, placenta, limbs and tail, etc.). Week 1 embryos were very small with little to no distinguishing characteristics. Week 2 embryos were larger with a small placenta, could be removed from the uterus, and a distinctive eyespot was observed. Week 3 embryos were large with almost fully developed characteristics. A 25 mg sample of the following tissue types was collected: the uterine arteries and veins, the uterus surrounding each individual embryo, the placenta if present, and the embryonic sac surrounding each embryo if available. Utensils were cleaned with bleach and ethanol between each type of tissue collection. If the embryo was early in development the whole embryo was used for DNA extraction or the embryo was cut in half to equal 25 mg. If the embryo was in a later developmental stage the heart of the embryo was removed and used in the extraction process. All embryo samples were screened for the presence of *B. microti* using the quantitative PCR protocols described in the following section.

### DNA extraction and PCRs

For all embryos and embryonic tissues, a 25 mg sample was collected as described in the previous section, DNA was extracted by hand using the Qiagen DNeasy Blood and Tissue kit following the manufacturer’s protocol (Qiagen, Valencia, CA, USA), and DNA concentration was analyzed using a spectrophotometer (Denovix Inc, New Castle County, Delaware, USA).

For adult samples, a 3 mm ear punch biopsy was collected from each mouse and DNA was extracted using the QIAcube HT DNA extraction system (Qiagen). DNA concentration was measured for each sample using a spectrophotometer (Denovix Inc). Each ear punch biopsy sample collected from adults was then tested in duplicate for the presence of *B. burgdorferi* using a quantitative PCR (qPCR) specific for a unique 69 bp segment of the *16S* rRNA gene: forward primer (5'-GGC GGC ACA CTT AAC ACG TTA G-3'), reverse primer (5'-GCT GTA AAC GAT GCA CAC TTG GT-3'), probe (6FAM-TTC GGT ACT AAC TTT TAG TTA A-MGBNFQ) [14]. Samples were run on a 7500 real-time PCR system (Applied Biosystems®, ThermoFisher Scientific, Waltham, WA, USA) using TaqMan Fast Advanced chemistry (ThermoFisher Scientific, Waltham, WA) and cycling conditions consisted of: 95 °C for 20 s, followed by 40 cycles of 95 °C for 3 s and 60 °C for 30 s. Because *B. burgdorferi* is an extracellular bacterium and primarily observed in tissues, we restricted our screening of *B. burgdorferi* to only adult ear punch biopsy samples.

For adult individuals, DNA from dried blood samples was extracted from FTA cards using the QIAcube HT DNA extraction system or by hand using the Qiagen DNeasy Blood and Tissue kit following the manufacturer’s protocol (Qiagen). DNA concentration was analyzed using a spectrophotometer (Denovix Inc). Embryos, embryonic tissues, and adult blood samples, were screened in duplicate for the presence of *B. microti* using a qPCR designed specifically for detecting a 104 bp section of the *18S* rRNA gene of *B. microti*: forward primer (5'-AAC AGG CAT TCG CCT TGA AT-3'), reverse primer (5'-CCA ACT GCT CCT ATT AAC CAT TAC TCT-3'), probe (6FAM-CTA CAG CAT GGA ATA ATG A-MGBNFQ) [15]. *Babesia microti* is primarily an intracellular erythrocytic protozoan and therefore we analyzed embryos, embryonic tissues, and adult dried blood samples for the presence of *B. microti*. Because blood samples could not be obtained from embryos, a 25 mg sample of heart tissue was also collected and analyzed from a subset of adult mice for direct comparison to embryos and embryonic tissues.

Average cycle threshold (CT) and quantity values were collected from each run and mean infection prevalence (number of infected individuals/total number of individuals) was calculated for each location. qPCR standards were constructed by separately cloning the aforementioned targeted regions of *B. burgdorferi* and *B. microti* into pUC57-Kan plasmids (GENEWIZ, Inc., South Plainfield, NJ, USA). A dilution series (10^6^–1 copy number dilutions) for each pathogen was developed by combining a single uninfected *I. scapularis* nymph (courtesy of the CDC) and a known amount of plasmid DNA followed by DNA extraction [14, 15]. Quantification and normalization of each sample were completed as previously described [14, 16, 17]; results are expressed as gene copies per picogram (pg) of total DNA.

*Babesia microti* positive samples, determined *via* qPCR, were then subjected to a standard PCR using a specific primer set to amplify 437 bp of the *B. microti* *18S* rRNA gene (forward PIRO-A: 5'-AAT ACC CAA TCC TGA CAC AGG G-3' and reverse PIRO-B: 5'-TTA AAT ACG AAT GCC CCC AAC-3') [18]. Amplification was performed using Platinum Superfi 2× PCR Master Mix (Invitrogen, ThermoFisher Scientific, Waltham, WA, USA) under the following conditions: 98 °C for 30 s, followed by 40 cycles of 98 °C for 10 s, 60 °C for 10 s, and 72 °C for 1 min, with a final elongation step of 72 °C for 5 min. PCR products were subjected to gel electrophoresis on a 1% agarose gel stained with ethidium bromide. Amplicons that produced bands of the correct size (~450 bp) were submitted for Sanger sequencing (Eurofins, Louisville, KY, USA) in both the forward and reverse directions. Consensus sequences were constructed and aligned with other orthologous *B. microti* sequences deposited in the GenBank database and previously described as human-infecting Clade 1 and nonhuman-associated Clade 2 and Clade 3 pathogens (AY144696, AY144701, AY144690; respectively) [19]. Alignments were completed by hand using BioEdit Sequencing Alignment Editor [20] and using the online Multiple Sequence Comparison by Log-Expectation Alignment (MUSCLE) program. Sequence diversity and a pairwise distance matrix were constructed using the maximum composite likelihood model in MEGA version 7.0 software [21].

### Statistical analyses

Infection prevalence was calculated for sex of adults, embryonic tissue type, and embryonic stage of development. Significant differences between the proportion of individuals infected and not-infected with *B. burgdorferi* were evaluated using a Fisher’s exact test. A Student’s t-test was used to compare the number of nymphs collected from April mice, individuals infected with *B. burgdorferi* and/or *B. microti* in April mice between males and females, between CT and BI, and to compare female infection to embryo infection overall and between trapping sessions. A nonparametric Kruskal-Wallis test was performed to assess differences in *B. microti* infection in the stages of embryonic development and the average number of embryos in each developmental stage. A pairwise Wilcoxon test was used to determine the variation between each stage of development. Finally, an analysis of variance (ANOVA) was completed to determine differences in parasitemia between the three stages of embryonic development. Geometric means of duplicate samples were used in all calculations.

## Results

All mice collected in April and only pregnant females (*n* = 6) and their embryos (*n* = 26) collected in July were necropsied and analyzed for the presence of *B. burgdorferi* and *B. microti* using qPCR. In the April sampling session, a total of 63 *P. leucopus* mice were collected from the Connecticut (CT) locations and 40 from the Block Island (BI) locations (Table 1). Of these, the combined proportion of mice with attached *I. scapularis* nymphs was 57.89% (*n* = 32 CT mice, *n* = 26 BI mice). Overall, 12.62% (CT = 9.53%; BI = 17.5%) of mice were infected with *B. burgdorferi* and 100% with *B. microti*. The average *B. burgdorferi* log mean copy number per pg total DNA (MCN) did not vary significantly between CT and BI (Table 1) or between infected males (*n*_CT_ = 4; *n*_BI_ = 5) and females (*n*_CT_ = 2; *n*_BI_ = 2). No significant differences in *B. microti* MCN were observed between CT and BI (Table 1) or between infected males (*n* = 55; MCN ± standard error = 5.25 ± 0.09) and females (*n* = 48; MCN = 5.20 ± 0.10).

**Table 1:** Log mean copy number per pg total DNA (MCN) calculations and standard errors (SE) for *Borrelia burgdorferi* and *Babesia microti* from Connecticut locations: Lake Gaillard (LG) and Old Lyme (OL) and Block Island locations: North Island (NI) and Rodman’s Hollow (RH) for all *Peromyscus leucopus* collected in April

		*B. burgdorferi*		*B. microti*	
	*n*	MCN	SE	MCN	SE
Connecticut	63	4.29	0.25	5.27	0.10
LG	27	3.95	0.29	5.43	0.18
OL	36	4.46	0.34	5.14	0.10
Block Island	40	4.35	0.20	5.17	0.10
NI	15	3.75	0.09	5.01	0.18
RH	25	4.59	0.17	5.27	0.11

**Fig. 2:**
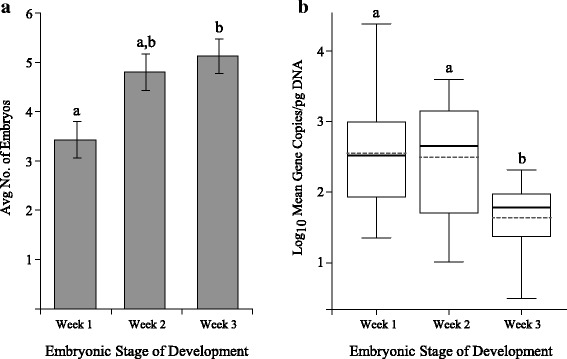
The average number of embryos in pregnant *Peromyscus leucopus* (**a**) and a box plot of the log mean copy number/pg DNA (MCN) of *Babesia microti* in embryos (**b**) in different stage of embryo development. Significantly fewer embryos were collected in Week 1 than in Week 3 (*P* = 0.0360). Parasitemia was significantly lower in Week 3 embryos compared to Week 1 and 2 (*P* < 0.0003 and *P* = 0.0022, respectively). Solid lines denote median values while dashed lines denote the means, different letters denote significant differences

Of the total small mammals sampled in 2016, 20 *P. leucopus* females were pregnant (*n*_CT_ = 12; *n*_BI_ = 8), including three and five mice collected on BI in April and July, respectively. All 12 pregnant females from CT were collected in April. One pregnant meadow vole (*Microtus pennsylvanicus*) was collected on BI in July and was included in some of the analyses. Four pregnant mice and the vole tested positive for *B. burgdorferi* (25.0%). All blood samples collected from adult pregnant females tested positive for the presence of *B. microti* via qPCR (100%) and no significant differences were observed between CT and BI females. The number of embryos per female (mouse only) ranged from 2–6 with an average of 4.5 embryos per female (Table 2; Additional file 1: Table S1). A significant difference among the average number of embryos was observed (Kruskal-Wallis *χ*^2^ = 8.13, *df* = 2, *P* = 0.0172). The average number of embryos in Week 1 (*n* = 3.4) was not significantly different from the number of embryos in Week 2 (*n* = 4.8; Wilcoxon test *P* = 0.0660), however there were significantly more Week 3 embryos compared to Week 1 (*n* = 5.1; Wilcoxon test *P* = 0.0360), but not compared to Week 2 (Wilcoxon test *P* = 0.4340; Fig. 2a). There were no significant differences observed in the average number of embryos between trapping sessions.

**Table 2:** The mean number of embryos at each embryonic stage of development, the total number of embryos in each stage, the number of those embryos infected with *Babesia microti*, and the infection prevalence of mouse embryos for each stage of embryonic development is presented

	Mean no. embryos	No. embryos	No. infected	% infected	MCN
Week 1	3.4	24	22	91.67	2.55
Week 2	4.8	24	21	87.50	2.50
Week 3	5.1	41	23	56.10	1.64
Total	4.5	89	66	74.16	2.22

See Additional file 1: Table S1 for detailed information of each individual female

A total of 89 mouse embryos were collected and tested for the presence of *B. microti*. Week 1 embryos showed the highest infection prevalence (91.67%), whereas Week 3 embryo infection was lowest (56.10%); the average infection prevalence among all embryos was 74.16% (Table 2). The average *B. microti* MCN was significantly different among the three stages of embryonic development (Kruskal-Wallis *χ*^2^ = 16.04, *df* = 2, *P* = 0.0003). Embryos from Week 1 (MCN = 2.55 ± 0.17) and Week 2 (MCN = 2.49 ± 0.18) displayed significantly higher parasitemia than embryos from the Week 3 stage of development (MCN = 1.64 ± 0.11; Wilcoxon test *P* = 0.0003 and *P* = 0.0022, respectively; Fig. 2b), but not between each other (Wilcoxon test *P* = 0.8948). Other reproductive tissue types (uterus, placenta, embryonic sac) also tested positive for *B. microti* (Additional file 2: Table S2), but due to the differences in embryo developmental stage not all tissue types could be collected at each of the three stages.

**Fig. 3:**
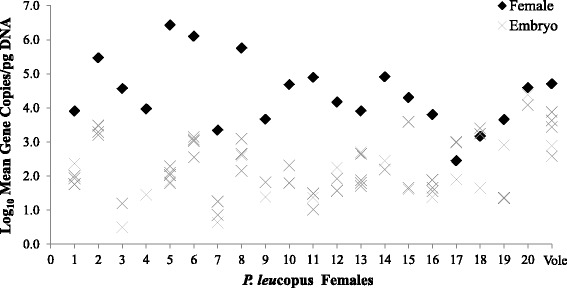
*Babesia microti* log mean copy number per pg of total DNA (MCN) comparing females to each of her infected embryos. Positions 1–20 are *Peromyscus leucopus* pregnant females; 1–16 were samples collected in April; 17–20 were collected in July, and the last position is of the single *Microtus pennsylvanicus* female (Vole) collected in July. Females (black diamonds) overall exhibited significantly higher parasitemia (MCN ± standard error, 4.405 ± 0.21) than their offspring (gray X; 2.291 ± 0.10; *P* < 0.0001)

Females displayed significantly higher *B. microti* parasitemia (*n* = 21; MCN = 4.40 ± 0.21) than their embryos (*n* = 71; MCN = 2.29 ± 0.10; *P* < 0.0001; Fig. 3). April females (*n* = 15; MCN = 4.67 ± 0.24) showed significantly higher parasitemia than pregnant females from July (*n* = 6 including the *M. pennsylvanicus* female; MCN = 3.73 ± 0.35; *P* = 0.0502). Conversely, July embryos (*n* = 20; MCN = 2.66 ± 0.22) exhibited a significantly higher MCN than embryos from April (*n* = 51; MCN = 2.15 ± 0.10; *P* = 0.0477). To assess differences in MCN among different sample types, we also compared heart tissue (*n* = 11, MCN 4.61 ± 0.24) and blood samples (*n* = 11, MCN = 5.00 ± 0.26) collected from a subset of adult mice and found that they did not differ significantly (*P* = 0.2757).

Sequences in the forward and reverse directions of the *18S *rRNA gene of *B. microti* were obtained for six individuals including each embryo, adult female uterine arteries and veins, uterus, placenta, and embryonic sac (total sequences = 76; Additional file 3: Figure S1). Consensus sequences were constructed and BLAST analysis (NCBI) confirmed all sequences to be strains of *B. microti* (XR_002459986 or AF028343). Most sampled sequences aligned nearly identically to the reference sequence for Clade 1 (AY144696), except for one sample of adult female arteries (2033-Arteries). The overall pairwise mean distance and sequence diversity was 0.005 for all embryonic samples (0.001 when 2033-Arteries was excluded) including the reference sequence for Clade 1 (AY144696) and 0.014 (0.011 when 2033-Arteries was excluded) when reference sequences from Clade 2 (AY144701) and Clade 3 (AY144690) were added. Unique *B. microti* sequences generated from this study have been deposited in GenBank (accession nos. MH221125, MH221126).

## Discussion

Our study demonstrates for the first time that transplacental transmission of *B. microti* occurs in wild, naturally-infected populations of *P. leucopus* and *M. pennsylvanicus* in Connecticut and on Block Island, RI, USA, with 74% transmission efficiency from mother to fetuses. Although studies have previously reported transplacental transmission of *Babesia canis canis* in canines [22, 23], *B. microti* in BALB/c laboratory mice [24], and humans [25, 26] only one other study has reported vertical transmission of *B. microti* occurring in naturally infected rodent populations [27]. Tolkacz and colleagues [27] reported vertical transmission of *B. microti* in two species of vole (*Microtus arvalis* and *M. oeconomus*) in Poland, finding 81% infection occurring in embryos and 90% in pups. Here, we report transplacental transmission in *P. leucopus* and a third species of vole, *M. pennsylvanicus*.

All individuals were infected with *B. microti* in April, in contrast to only 12.6% of individuals infected with *B. burgdorferi*. These mice could have been infected by nymphal *I. scapularis* in the spring or could have survived and maintained the infection through the winter months (chronic infection). Because nymphal *I. scapularis* were found on 50.8% of mice collected in CT and 65.0% of mice collected on BI, it is possible these mice all became infected by questing nymphs once they emerged from their winter burrows. However, because a higher proportion of nymphal *I. scapularis* are typically infected with *B. burgdorferi* (22.37% average infection prevalence) than *B. microti* (9.53% average infection prevalence) in the New England area (comparing 10 studies and 9335 nymphal *I. scapularis* samples) [6], the higher *B. microti* infection prevalence in mice requires an alternative explanation. A combination of vertical transmission and chronic infection with *B. microti* through the winter months would amplify *B. microti* even before most ticks emerged from diapause, typically in mid to late May [6], providing a transmission advantage compared to *B. burgdorferi*. The duration of *B. microti* infection in naturally infected (*via* tick vector) *P. leucopus* is unclear; however, chronic infection has been shown to persist in laboratory *P. leucopus* for seven months and possibly longer [9, 10, 28, 29]. Chronic infection of *B. microti* was also observed for approximately five months in naturally infected voles from Russia [30]. In these ways mice infected over the transmission season (April-July) could remain infected until the following spring, when transplacental transmission could further increase early-season transmission. Resistance to re-infection is unknown in *Peromyscus*; consequently, mice could either become re-infected or *B. microti* could become reactivated every transmission season resulting in chronic infection and an increase of the pathogen in the population *via* vertical transmission. Reactivation of certain pathogens has been shown to occur during stressful life events (i.e. migration in birds) [31, 32]. Seasonal physiological stress (i.e. cold temperatures during winter months) may allow for reactivation of *B. microti* and provide an additional explanation for prolonged infection in wild *P. leucopus*.

The relative timing of insemination and infection in the mother may be important determinants of the survivorship and infection outcome of fetuses [24, 33]. The breeding season of *P. leucopus* in our study region starts around March, while nymphal tick activity typically starts in April or May, although both events are highly dependent on seasonal variation in temperature [34]. Furthermore, peak *B. microti* parasitemia in *P. leucopus* occurs 14 days after infection *via* tick bite [35]. Given the differences in mouse and tick life cycles, females captured in April were more likely inseminated before *B. microti* infection *via* ticks. The lower average number of Week 1 compared to Week 3 embryos may be explained by the higher likelihood of reabsorption of some or all embryos when the female is inseminated simultaneously or before infection. Reabsorption of embryos was suggested in a study using BALB/c laboratory mice which found that females inseminated in the acute phase of *B. microti* infection (0–12 days) did not produce any offspring [24]. Reabsorption of fetuses when mice are infected early in the gestation period has also been observed in other pathogens, such as *Neospora caninum* in BALB/c laboratory mice [33]. A significantly higher number of Week 3 embryos with significantly lower parasitemia compared to embryos in earlier stages of development (Week 1 and 2) was observed. This may also indicate that females became infected with *B. microti* after insemination or during later stages of gestation and the pathogen had not fully disseminated to all embryos when the female was euthanized. Experimental manipulation of the timing of infection and insemination is required to fully characterize the affect *B. microti* may have on developing fetuses and pathogen persistence.

Females exhibited significantly higher parasitemia compared to their offspring. Although the different tissues investigated could partially account for this difference, we found no significant differences in MCN in a subset of samples of adult heart tissue and blood. Decreased parasitemia in embryos may thus be due to limited placenta permeability. Although the placenta is known to function as a barrier to potentially harmful microbes and pathogens [36], multiple parasites and pathogens have developed ways of evading the placental barrier to infect a fetus, including another apicomplexan, *Plasmodium* spp. [37], as well as *Toxoplasma gondii* [38], *Trypanosoma* spp. [39], and several nematodes [40, 41]. Similar to *Plasmodium* spp*.*, *B. microti* sporozoites invade host red blood cells and are able to breach the placental barrier to infect a female’s offspring [42]; exactly how these pathogens are able to diffuse through the placenta is unknown.

Tolkacz and colleagues [27] found different genotypes of *B. microti* in female voles and their offspring, although the mechanisms for these differences were not explained. This was not the case in our study, with sequences from *P. leucopus* and *M. pennsylvanicus* revealing high similarity between females and fetuses and overall. Low genome-wide sequence diversity was also observed in other studies [43, 44]. Some studies suggest that *B. microti* represents a rich species complex consisting of three distinct clades in the United States using the *18S* rRNA gene; however genetic diversity within each clade is relatively invariant [19]. Strains in Clade 1 are found associated with human-biting *Ixodes* species, locations known to be endemic for human babesiosis, and exhibit the least amount of genetic diversity. Whereas strains in Clade 2 were isolated from carnivores and those in Clade 3 were found to infect rodents; neither Clade 2 nor 3 have been found to infect humans. Sequences from this study most closely aligned with the reference sequence from Clade 1 and these sequences showed a low sequence diversity of 0.001. One of our sequences (2033-Arteries) exhibited higher diversity than predicted by previous studies; this might have occurred due to multiple infections or interference from other pathogens during Sanger sequencing (resulting in a noisy chromatograph) and most likely does not represent a unique *B. microti* species.

Our field-based results indicate that transplacental transmission of *B. microti* is a potentially important pathway for infection in wild rodent species, *P. leucopus* and *M. pennsylvanicus*, which may partially explain *B. microti* emergence and geographic expansion. In endemic areas, transplacental transmission may enhance early season amplification of *B. microti*, contributing to higher infection prevalences in both hosts and vectors. Additional enhancement mechanisms have been described, including increased transmission of *B. microti* to ticks feeding on hosts coinfected with *B. burgdorferi* based in field and laboratory studies [6, 11, 35] and amplification driven by tick aggregation, as shown in an empirically-informed model [45]. Transplacental transmission in natural populations may also facilitate *B. microti* maintenance and spread in small rodent populations in areas with limited *I. scapularis* occurrence. For instance, in areas of Alaska, California, Colorado, Florida, Maine, and Montana that lack *I. scapularis*, small rodent populations (voles, shrews, cotton rats, other *Peromyscus* spp.) were infected with both human-infecting and non-human-associated strains of *B. microti* [46–50]. Some evidence suggests that nidicolous ticks (e.g. *I. angustus*, *I. muris*, *I. spinipalpis*) or other exophilic ticks (*I. pacificus*) may contribute moderate enzootic persistence in certain areas [48] in a ‘cryptic cycle’, but vertical transmission may also play a role. Irrespective of the maintenance mechanism in the enzootic cycle, transmission to humans and emergence would only occur when *I. scapularis* (a ‘bridge’ vector to humans) invades a region where *B. microti* had been enzootically maintained, as has been shown for *B. burgdorferi* [49, 51].

To confirm the relevance of transplacental and vertical transmission in *B. microti*, studies in a laboratory setting are required to specifically quantify the relative efficiencies of *B. microti* transmission routes, including tick-to-host transmission, vertical transmission in relation to insemination and infection timing, chronic infection, and xenodiagnoses and transmission efficiency of infected offspring. More extensive field studies quantifying the role of vertical transmission and chronic infection of *B. microti* should then be conducted to assess how these transmission pathways influence this pathogen’s persistence and transmission, in comparison to *B. burgdorferi*. As winter temperatures in these areas continue to increase each year [52], overwinter survival of mice may increase [53, 54], further enhancing the early season advantage of *B. microti* through chronic infection and vertical transmission.

## Conclusions

We demonstrate that transplacental transmission of *B. microti* occurs with high efficiency in wild rodent populations in New England. This non-vector mediated transmission mode may result in significant pathogen amplification, in particular if combined with chronic infection in the host and increased overwintering survival in warming climates.

## Additional files

Additional file 1: **Table S1.** Information for each pregnant female and infection status of her embryos collected in April and July, 2016. Site: LG, Lake Gaillard; OL, Old Lyme; RH, Rodman’s Hollow; NI, North Island. Age of embryos: W1, Week 1; W2, Week 2; W3, Week 3. (DOCX 19 kb)

Additional file 2: **Table S2.** *Babesia microti* infection prevalence in reproductive tissues (ES, embryonic sac). (DOCX 14 kb)

Additional file 3: **Figure S1.** *Babesia microti* sequence alignment from embryos and tissues of the *18S* rRNA gene for six females (labeled by a four digit identification number) including three reference sequences representing the three *Babesia* clades (Clade-1, AY144696; Clade-2, AY144701; Clade-3, AY144690). (DOCX 136 kb)

## Abbreviations

BI: Block Island, Rhode Island
CT: Connecticut
LG: Lake Gaillard
MCN: log mean copy number/pg of DNA
NI: North Island
OL: Old Lyme
qPCR: Quantitative polymerase chain reaction
RH: Rodman’s Hollow
