# Supplementary material for: Transplacental transmission of tick-borne Babesia microti in its natural host Peromyscus leucopus
Source: Parasit Vectors. 2018 May 4;11:286. doi: 10.1186/s13071-018-2875-8 (PMC5935994; doi:10.1186/s13071-018-2875-8)
Supplement: Supplementary file 1 — Table S1. Information for each pregnant female and infection status of her embryos collected in April and July, 2016. Site: LG, Lake Gaillard; OL, Old Lyme; RH, Rodman’s Hollow; NI, North Island. Age of embryos: W1, Week 1; W2, Week 2; W3, Week 3. (DOCX 19 kb) [file 13071_2018_2875_MOESM1_ESM.docx]

Supplemental Table 1. Information for each pregnant female and infection status of her embryos collected in April and July, 2016. Site: LG = Lake Gaillard, OL = Old Lyme, RH = Rodman’s Hollow, NI = North Island. Age of embryos: W1 = Week 1, W2 = Week 2, W3 = Week 3.

| ID | State | Site | Total no. embryos | Embryos infected | Age of embryos | % Infected | MCN/pg DNA | Std dev | SE |
| --- | --- | --- | --- | --- | --- | --- | --- | --- | --- |
| 1 | CT | LG | 4 | 4 | W1 | 100.00% | 2.02 | 0.25 | 0.13 |
| 2 | CT | LG | 4 | 4 | W2 | 100.00% | 3.36 | 0.13 | 0.07 |
| 3 | CT | LG | 5 | 2 | W3 | 40.00% | 0.85 | 0.50 | 0.35 |
| 4 | CT | LG | 6 | 1 | W3 | 16.67% | 1.45 | NA | NA |
| 5 | CT | LG | 6 | 5 | W3 | 83.33% | 2.01 | 0.20 | 0.09 |
| 6 | CT | LG | 5 | 5 | W2 | 100.00% | 2.97 | 0.24 | 0.11 |
| 7 | CT | OL | 5 | 3 | W3 | 60.00% | 0.92 | 0.31 | 0.18 |
| 8 | CT | OL | 5 | 5 | W1 | 100.00% | 2.63 | 0.33 | 0.15 |
| 9 | CT | OL | 5 | 2 | W3 | 40.00% | 1.60 | 0.31 | 0.22 |
| 10 | CT | OL | 6 | 3 | W3 | 50.00% | 2.14 | 0.30 | 0.17 |
| 11 | CT | OL | 5 | 3 | W2 | 60.00% | 1.29 | 0.25 | 0.14 |
| 12 | CT | OL | 3 | 3 | W3 | 100.00% | 1.91 | 0.35 | 0.20 |
| 13 | RI | RH | 6 | 6 | W2 | 100.00% | 2.23 | 0.48 | 0.20 |
| 14 | RI | RH | 2 | 2 | W1 | 100.00% | 2.32 | 0.18 | 0.13 |
| 15 | RI | NI | 4 | 3 | W2 | 75.00% | 2.28 | 1.14 | 0.65 |
| 16 | RI | RH | 5 | 4 | W3 | 80.00% | 1.62 | 0.21 | 0.10 |
| 17 | RI | RH | 4 | 3 | W1 | 75.00% | 2.62 | 0.63 | 0.36 |
| 18 | RI | RH | 3 | 3 | W1 | 100.00% | 2.76 | 0.97 | 0.56 |
| 19 | RI | RH | 3 | 3 | W1 | 100.00% | 1.87 | 0.89 | 0.52 |
| 20 | RI | NI | 3 | 2 | W1 | 66.67% | 4.23 | 0.21 | 0.15 |
| Vole | RI | RH | 5 | 5 | W2 | 100% | 3.28 | 0.54 | 0.24 |
