# Supplementary material for: Transplacental transmission of tick-borne Babesia microti in its natural host Peromyscus leucopus
Source: Parasit Vectors. 2018 May 4;11:286. doi: 10.1186/s13071-018-2875-8 (PMC5935994; doi:10.1186/s13071-018-2875-8)
Supplement: Supplementary file 2 — Table S2. Babesia microti infection prevalence in reproductive tissues (ES, embryonic sac). (DOCX 14 kb) [file 13071_2018_2875_MOESM2_ESM.docx]

Supplemental Table 2. *Babesia microti* infection prevalence in reproductive tissues, ES = embryonic sac.

|  | Uterus | Placenta | ES | Embryos |
| --- | --- | --- | --- | --- |
| Week 1 | 100.00% | NA | NA | 91.67% |
| Week 2 | 88.00% | 100.00% | NA | 87.50% |
| Week 3 | 62.96% | 75.00% | 63.33% | 56.10% |
