# Supplementary material for: Transplacental transmission of tick-borne Babesia microti in its natural host Peromyscus leucopus
Source: Parasit Vectors. 2018 May 4;11:286. doi: 10.1186/s13071-018-2875-8 (PMC5935994; doi:10.1186/s13071-018-2875-8)
Supplement: Supplementary file 3 — Figure S1. Babesia microti sequence alignment from embryos and tissues of the 18S rRNA gene for six females (labeled by a four digit identification number) including three reference sequences representing the three Babesia clades (Clade-1, AY144696; Clade-2, AY144701; Clade-3, AY144690). (DOCX 136 kb) [file 13071_2018_2875_MOESM3_ESM.docx]

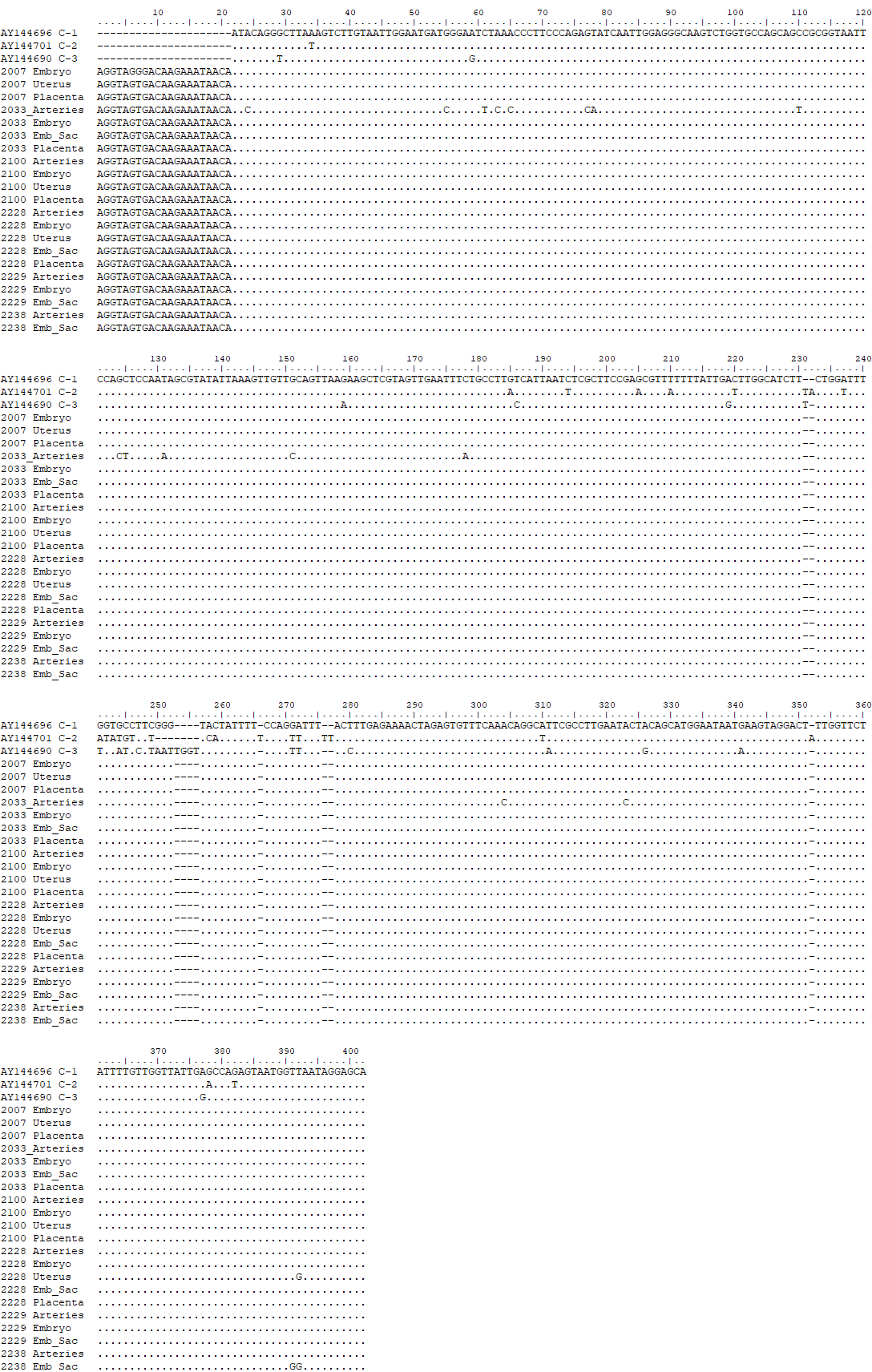


Supplemental Figure 1. *Babesia microti* sequence alignment from embryos and tissues of the 18S rRNA gene for six females (labeled by a four digit identification number) including three reference sequences representing the three *Babesia* clades (Clade-1 AY144696; Clade-2 AY144701; Clade-3 AY144690).
